# Challenges and perspectives for naming lipids in the context of lipidomics

**DOI:** 10.1007/s11306-023-02075-x

**Published:** 2024-01-24

**Authors:** Michael Witting, Adnan Malik, Andrew Leach, Alan Bridge, Lucila Aimo, Matthew J. Conroy, Valerie B. O’Donnell, Nils Hoffmann, Dominik Kopczynski, Franck Giacomoni, Nils Paulhe, Amaury Cazenave Gassiot, Nathalie Poupin, Fabien Jourdan, Justine Bertrand-Michel

**Affiliations:** 1 Metabolomics and Proteomics Core, Helmholtz Zentrum München, Ingolstädter Landstraße 1, 85764 Neuherberg, Germany; 2 Chair of Analytical Food Chemistry, TUM School of Life Sciences, Technical University of Munich, Maximus-von-Imhof-Forum 2, 85354 Freising-Weihenstephan, Germany; 3 European Molecular Biology Laboratory, European Bioinformatics Institute (EMBL-EBI), Wellcome Genome Campus, Hinxton, Cambridge, CB10 1SD UK; 4 SIB Swiss Institute of Bioinformatics, Centre Medical Universitaire, 1211 Geneva 4, Switzerland; 5 Division of Infection and Immunity, Systems Immunity Research Institute, School of Medicine, Cardiff University, Cardiff, CF14 4XN UK; 6 Institute for Bio- and Geosciences (IBG-5), Forschungszentrum Jülich GmbH, 52425 Jülich, Germany; 7 Institute for Analytical Chemistry, Universität Wien, Währingerstrasse 38, 1090 Vienna, Austria; 8 Université Clermont Auvergne, INRAE, UNH, Plateforme d’Exploration du Métabolisme, MetaboHUB Clermont, Clermont-Ferrand, France; 9 MetaboHUB, National Infrastructure of Metabolomics and Fluxomics ANR-11-INBS-0010, 31077 Toulouse, France; 10 Singapore Lipidomics Incubator, Life Sciences Institute, and Precision Medicine TRP, Yong Loo Lin School of Medicine, National University of Singapore, Singapore, Singapore; 11 UMR1331 Toxalim, Université de Toulouse, INRAE, ENVT, INP-Purpan, UPS, Toulouse, France; 12 I2MC, Inserm U1297, Université de Toulouse, Toulouse, France

**Keywords:** Lipidomic, Anotation, Identification, Interoperability

## Abstract

**Introduction:**

Lipids are key compounds in the study of metabolism and are increasingly studied in biology projects. It is a very broad family that encompasses many compounds, and the name of the same compound may vary depending on the community where they are studied.

**Objectives:**

In addition, their structures are varied and complex, which complicates their analysis. Indeed, the structural resolution does not always allow a complete level of annotation so the actual compound analysed will vary from study to study and should be clearly stated. For all these reasons the identification and naming of lipids is complicated and very variable from one study to another, it needs to be harmonized.

**Methods & Results:**

In this position paper we will present and discuss the different way to name lipids (with chemoinformatic and semantic identifiers) and their importance to share lipidomic results.

**Conclusion:**

Homogenising this identification and adopting the same rules is essential to be able to share data within the community and to map data on functional networks.

## Introduction

Lipids are a heterogenous group of compounds that play important roles in regulating complex biological processes (e.g., cell signalling, energy storage, and membrane formation) and, thus, in life itself. An ever-increasing number of novel lipids with a wide range of interesting and potentially beneficiary properties are being identified from sources such as plants, fungi and microorganisms. Lipids are thus clearly of critical interest to the scientific community. However, many biologists lack the detailed expertise and knowledge to fully understand and appreciate the many complex and subtle aspects of lipids and their biology, and in particular the many nuances associated with the accurate representation of chemical structures. A further challenge is that the same lipid will often be referenced by multiple names and synonyms in the scientific literature and in databases. And moreover depending on the analytical technique used we will not have access to the same detail of structural information or the same level of annotation of the studied lipid. Indeed, structural description of lipids from typical lipidomics experiments is way more superficial than it would be required for detailed biological investigations. While for example, biosynthetic pathways of major fatty acids are well established in many organisms, even down to location and stereochemistry of double bonds, this information cannot be determined once they are bound in complex lipids, especially if they are not previously cleaved or derivatized. This complexity and ambiguity are a significant obstacle and can lead to wasted effort, inaccurate results and misleading conclusions. Biochemical interpretation of lipidomics results is hampered by these factors. Therefore, there is a need to develop reliable and trusted resources that provide correct information about lipid molecules at the level of structural characterization in line with the capabilities of possible with the selected analysis method, thereby delivering a solution to many of these challenges. New analytical approaches such as electron activated dissociation (Baba et al., 2017) (EAD) or ozone induced dissociation (Brown et al., 2011) (OzID) will in the future facilitate analysis and determination of more structural details. Until these approaches, tools and resources become routinely available, great care needs to be taken to avoid overreporting and incorrectly annotating lipidomic results, for the interpretation of such results. Here we will recall the challenges for lipid identification, present the available identifiers (chemoinformatics and semantic) (Koistinen et al., 2023), the difficulties in mapping lipid data into functional networks, i.e. metabolic network and finally we will propose suggestion for harmonizing the reporting of lipids. We aimed to include examples of databases and tools that would concur with the ideas we have conveyed in this article, but did not aim to cover all possible options. The choice of tools is based either on individual usage experiences or active involvement by the authors in the development of said tools. There is no claim to exhaustively cover all tools.

## Challenges for reporting of lipid identification

### One lipid, one name?

In lipidomics different disciplines collide: biochemistry, analytical chemistry and bioinformatics. Each with a very domain-specific language and terminology. One particular molecule might be known under different names to different scientists or different names might be used in different disciplines. One simple example is oleic acid, which is also referred to as (Z)-octadec-9-enoic acid or many other names (Table 1). For example, a biochemist might talk about oleate, analytical chemists using GC–MS might state that oleic acid has been identified. Though from a chemistry point of view, these two are technically two separate entities (acid vs. conjugate base), both might refer to the same molecule. Different possibilities to represent these molecules exist (Table 1).

**Table 1:** Example of the different names and identifiers given to oleic acid


Name:	Oleic acid
Species level:	FA 18:1
DB position level:	FA 18:1(9)
Full Structural level:	FA 18:1(9Z)
IUPAC Name:	(Z)-octadec-9-enoic acid
Synonyms:	(9Z)-Octadecenoic acid, 18:1 n-9, 18:1Δ9cis, C18:1 n-9, cis-9-octadecenoic acid, cis-Δ9-octadecenoic acid, …
Formula:	C18H34O2
SMILES:	CCCCCCCC\C=C/CCCCCCCC(O)=O
InChI:	1S/C18H34O2/c1-2-3-4-5-6-7-8-9-10-11-12-13-14-15-16-17-18(19)20/h9-10H,2-8,11-17H2,1H3,(H,19,20)/b10-9-
InChIKey:	ZQPPMHVWECSIRJ-KTKRTIGZSA-N
ChEBI:	16196
HMDB:	HMDB0000207
LipidMaps:	LMFA01030002
SwissLipids:	000000418^a^
KEGG:	C00712

^a^Note that the SwissLipids ID refers to the anion (9Z)-octadecenoate, which maps to ChEBI:30823

Different databases list this and other molecules, but they might be cross-referencing each other. In summary, multiple (correct) synonyms exist for different molecules and also multiple entries across different databases. In the case of a relatively simple molecule such as oleic acid conflicts might be easily (and even automatically) resolved. However, in the case of complex lipids this situation becomes even more complicated. For example, in HMDB (Wishart et al., 2022) lists we can find 66 different synonyms for PC 16:0/18:1(9Z). IUPAC names are also getting more complicated, e.g. (2-{[(2R)-3-(hexadecanoyloxy)-2-[(9Z)-octadec-9-enoyloxy]propylphosphono]oxy}ethyl)trimethylazanium for PC 16:0/18:1(9Z). However, this name is referring to a fully characterized chemical structure, with known sn-positions and stereoisomer configuration of fatty acyls chain, as well as the location of the double bond on the second fatty acyl chain. Depending on the analytical method employed, the level of detail required for full structural characterization may not be realizable leading to a discrepancy between analytical results and potential biochemical knowledge.

### Analytical power and structural resolution

Structural resolution, which corresponds to the structural detail of given lipid species (Koelmel et al., 2017) depends on the complexity of the studied lipid and on the analytical technique used. A simple lipid such as a sterol (and its derivatives), fatty acid (and its derivatives) or sphingoid base could be easily characterized by chemical or spectrometric methods, pure standards are available and their mass spectra are also known, enabling identification even down to full structural detail. Complex lipids such as phospholipids, sphingolipids or triacylglycerides may be only partially characterizable. Chromatographic separation coupled with MS/MS detection further helps to gather evidence to determine more lipid structural characteristics. However, it is sometimes not straightforward to obtain their stereochemistry and isomerism, since there is a lack of enantiomerically-pure standards and chiral methods are often not applied (Köfeler et al., 2021). But for the vast majority of lipids such as phospholipids and sphingolipids, where it is complicated to annotate to a single lipid species, different degrees of structural resolution will be proposed. If we consider the phosphatidylcholine family, most often analytical chemists will be able to propose the simplest structural resolution, comprising the nature of the polar head, the number of carbon and double bond equivalents (DBE), for example, PC 34:2. If MS^2^ fragmentation is used, the fatty acid constituents can be designated (PC 16:0_18:2). More rarely positional isomers (PC 18:0/18:2) and almost never the double bond position (PC 16:0/18:2(10,12)), double bond Cis/Trans (PC 16:0/18:2(10E,12Z)) or stereochemistry (PC 16:0/18:2(10E,12Z)[R]) can be determined. These different levels of structural resolution are now very well described and the international community has agreed to use these names rigorously in all lipidomic datasets (Liebisch et al., 2020). Considering our simple oleic acid (Table 1), 3 different structural resolution levels can be designated: FA 18:1, FA 18:1(9) or FA 18:1(9Z). Due to the many subtleties of lipid nomenclature, old dialects and conventions, as well as the newer, consolidated shorthand nomenclature as a common lingua-franca, automation to aid in the conversion and validation of lipid names is needed.

## Which identifiers are available?

Over the last few decades many communities have attempted to identify molecules more rigorously through informatic or semantic identifiers. Each identifier has different advantages and disadvantages, which are discussed below.

### Chemoinformatic identifiers

The chemical structure is the most unique identifier of a lipid. A set of possible representations of molecules is available in the literature, allowing these chemical structures to be stored and used in computer systems, with associated chemoinformatic libraries and tools. These structure representations are commonly enumerated with one- (1D) or two-dimensional (2D) linear notations and molecular file concepts (Faulon & Bender, 2010). The specifications of each format covers organic chemistry and allow the description of structures from a wide variety of chemical families but with different levels of information (e.g., double bond localisation). In the main lipid databases, in addition to having a 2D visual molecular representation, information on each fully resolved lipid structure is available in the Simplified Molecular Input Line Entry Specification (SMILES) and International Chemical Identifier (InChI) formats.

*SMILES* is a proprietary format developed by Daylight Chemical Information Systems (https://www.daylight.com/). Although it is widely used by the chemical community, its adoption as a gold standard has been slowed by the fact that its specifications remain proprietary. Academic initiatives led by the Blue Obelisk community, for example, and its OpenSMILES (http://opensmiles.org/) format have opened up the format specifications to the chemical and bioinformatics community.

*InChI* and its hashed form InChikey are open formats developed by the non-profit InChI Trust (https://www.inchi-trust.org/) and the International Union of Pure and Applied Chemistry (IUPAC) (Heller et al., 2015). This textual identifier is a standard encoding for molecular information and a means of facilitating the retrieval of this information from databases. Its continued development provides solutions to such complex issues as the management of tautomers (Dhaked et al., 2020).

Computed from the computer-readable representation of a chemical structure, the InChI is encoded by a string including a prefix (“InChI = 1/”), several layers (e.g., empirical formula, hydrogens, charge or protonation/deprotonation layers) in the core parent structure and additional features (e.g., stereochemistry, isotopic or FixedH layers) (Heller et al., 2015).

The use of SMILES and InChI format in lipidomic annotation is promising. But due to the lack of fully resolved structural information from chromatography-mass spectrometry, some common representations in the structure of lipids cannot be well supported. For example, the position of a double bond and a fatty acid on the backbone cannot be left undetermined due to the mandatory presence of the absolute bond connectivity definition layer (Koelmel et al., 2017). With this limitation, SMILES or InChI string can be computed for the cholesteryl linoleate (a (9Z,12Z)-stereoisomer of cholesteryl octadeca-9,12-dienoate) but not for instance for CE 18:2, a cholesterol ester in which the acyl group contains 18 carbons in total and 2 double bonds at undetermined positions. However, a sum composition in the form of a chemical formula can be determined. Due to this reason other identifiers are required that support structural ambiguity.

### Semantic identifiers

Semantic identifiers refer to constant identifiers of concepts in a databas that are connected with each other via semantic relationships. In lipidomics, such databases can either store the full lipid structures with all structural details or higher level structures, which only encode common traits of members of the same lipid class or category. This taxonomic, hierarchical relationship is important for the reporting of lipid identities at the level of structural resolution that is supported by the applied analytical method. However, these databases often also incorporate aspects of ontologies, whereby the relationships between molecular entities or classes of entities and their parents and/or children, as well as their subcomponents, such as fatty acyls, are specified. This ontological structure is very relevant in the case of lipids to ensure classification at the appropriate structural level.

The *LIPID MAPS* resource (https://lipidmaps.org/databases) has been instrumental in providing a classification for lipids (Fahy et al., 2009) which is used by the worldwide lipidomics community. Lipids are classified into eight main categories: Fatty acyls, Glycerolipids, Glycerophospholipids, Sphingolipids, Sterols, Prenols, Saccharolipids and Polyketides. They are further classified into one or more of several sub-classes as appropriate. LIPID MAPS has provided a standardised nomenclature and shorthand notation (Liebisch et al., 2020) for lipids at varying levels of chemical characterisation.

The main LIPID MAPS Database, LMSD (Sud et al., 2007) contains nearly 50,000 structures and annotations of biologically relevant lipids, both manually curated from literature, experiment and brought in from other resources, and also computationally generated using commonly occurring acyl chains (https://www.lipidmaps.org/databases/lmissd/overview). This can be queried in a number of ways, including name, shorthand, InChiKey and structure, but also from a list of precursor ion masses.

COMP_DB contains over 60,000 ‘bulk’ lipids for (phospho)glycerolipids, fatty acyls, sphingolipids and sterols. These ‘bulk’ lipid species indicate the number of carbons and number of DBE, but not chain positions or double bond regiochemistry and geometry. It might be particularly useful where fragmentation is not available and data are insufficient to characterise a lipid fully. It is also of use in querying lipid classes which are less well represented in LMSD due to the paucity of full-structural characterisation, for example of the betaine lipid family.

Chemical Entities of Biological Interest (ChEBI) is a high quality, manually curated, open access database and ontology of information about small molecular entities. The molecular entities in question are either naturally occurring molecules or synthetic compounds used to intervene in the processes of living organisms (Degtyarenko et al., 2007). ChEBI currently contains 29,800 lipid entries (February 2023) and creates for each distinct lipid structure a stable and unique identifier (ChEBI ID), which is used by multiple other resources to unambiguously identify that specific compound. Each distinct lipid structure, whether it be neutral, ion, tautomer, enantiomer, salt, hydrate etc. will have a unique ChEBI ID. For example, oleic acid has CHEBI: 16196), its ionised form oleate has CHEBI: 30823 interconnected via the ontology by a bidirectional conjugate acid/base relationship. ChEBI also serves as a manually curated source of chemical structures, nomenclature (synonyms, ChEBI recommended names, IUPAC names, and international nonproprietary names), metabolite species information, and database cross references (Matos et al., 2010). ChEBI provides database cross-reference links to 65 different domain-specific resources by manual synonym matching and InChI key mapping via UniChem (Chambers et al., 2013). These include links to genomic (Gene Ontology), proteomic (PDBe, UniProt), metabolomics (MetaboLights), immunological (IEDB), toxicological (ACToR), pathway (Reactome), reaction (Rhea), system model (BioModels) databases, together with the broader scientific literature (Europe PMC). Having such a widely used standard representation for lipid data helps drive the data integration of diverse data types via common and related chemical entities. ChEBI currently contains 160,800 entries of which 60,400 (February 2023) are manually curated and new chemical entities are continuously being added to the database by its growing user community via the submissions tool. A key feature of ChEBI is that it provides a hierarchical ontological classification for lipids, whereby the relationships between lipid entities or classes of lipids are specified. The ChEBI ontology is subdivided into two main sub-ontologies: a molecular structure ontology where lipids are classified according to composition and structure (e.g., steroids, fatty acids etc.) and role ontology which classifies lipids on the basis of their role within a chemical context (e.g., emulsifier), biological context (e.g., enzyme inhibitor), or application (its intended use by a human e.g., antineoplastic agent, pesticide etc.). As an example of the structure ontology, the fatty acid arachidonic acid (5Z,8Z,11Z,14Z-icosatetraenoic acid) is classified as an “icosa-5,8,11,14-tetraenoic acid”, which is itself indicated as an “icosatetraenoic acid”. The ChEBI ontology is widely used for knowledge-based automated reasoning in systems biology and bioinformatics. The ontology is also semantically integrated with many other biological ontologies, for example, the Gene Ontology uses ChEBI for all its chemistry-related terms (Hill et al., 2013). All of the information and data in ChEBI is freely available and downloadable without restriction.

In contrast to LipidMaps ChEBI also stores partial structures, which is important for correct reporting of lipid identification. These structures are linked by relations defined in the ontology, e.g. PC(18:0_18:1) (CHEBI:167255) “is a” phosphatidylcholine 36:1 (CHEBI:66857). New entries are constantly added and integrated.

*SwissLipids* (www.swisslipids.org) is a free knowledge base aiming to facilitate the interpretation of experimental datasets and integrate them with prior biological knowledge. This resource was created through an iterative process in which prior knowledge of lipid structures and metabolism curated from peer-reviewed literature was used to generate an in silico library of all theoretically possible structures that could be present in natural lipidomes (Aimo et al., 2015). For this, characterised lipid structures and their lipid classes are annotated using the ChEBI ontology (www.ebi.ac.uk/chebi/) (Hastings et al., 2016). SMILES representations of modular lipid classes were then combined with the different curated fatty tails (fatty acyls and fatty alcohols) using the Java application SMILIB v2.0, resulting in an in silico library. This library is organized in a hierarchical classification consistent with the lipid notation described above, ranging from complete definition (isomers) to general definition (classes) (Liebisch et al., 2013, 2020). To help identify lipids, all annotations include lipid nomenclature and human readable descriptions, SMILES representations, molecular formula, mass, and InChI and InChI keys where applicable, identifiers from ChEBI, HMDB and LPIPID MAPS, including ChEBI identifiers for corresponding parent classes and structural components. SwissLipids provides a simple interface searchable by these descriptors, a menu to browse by structural classification paralleling that of LIPID MAPS, and a menu that allows searching with MS outputs in the hierarchical classification by selecting the lipid class and sum formula, that links to biological knowledge such as enzymes, as follows:

$${\text{PC}}\quad 38:4 \to {\text{PC}}\quad 18:0\_20:4 \to {\text{PC}}\quad 18:0/20:4 \to {\text{PC}}\quad 18:0/20:4\left( {5{\text{Z}},8{\text{Z}},11{\text{Z}},14{\text{Z}}} \right) \to {\text{PLA}}2{\text{G}}4{\text{A}}$$

The Swisslipids library contains almost 600,000 lipid structures (known and theoretical) belonging to over 500 lipid classes, each enriched with information on lipid components, metabolism (described using the Rhea knowledgebase (www.rhea-db.org) (Morgat et al., 2020), which itself is based on ChEBI (Bansal et al., 2022), and enzymes (using the UniProt Knowledgebase, UniProtKB (www.uniprot.org), with supporting links to primary literature. Like SwissLipids, UniProtKB uses Rhea as its main vocabulary for catalytic activity (Morgat et al., 2020), and the ChEBI ontology for small molecules (Coudert et al., 2023) (UniProt Consortium, 2021). This means UniProtKB can now be searched with lipid names, chemoinformatics identifiers, as well as ChEBI IDs, to retrieve all lipid interacting proteins, including enzymes and transporters. SwissLipids, Rhea and UniProtKB, all consistently use ChEBI IDs corresponding to the major microspecies at pH 7.3 in order to balance all reactions by mass and charge, and ensure all reactions are unique.

## Conversion of identifiers

Each presented database represents a valuable resource for lipid reporting and often covers a specific aspect of lipid biochemistry. In order to leverage the full potential of their combination solutions for the conversion of identifiers into each other are required. Different software tools for the conversion of lipid shorthand nomenclature or conversion of database IDs have been developed.

LipidLynxX provides the possibility to convert, cross-match, and link various lipid annotations to the tools offering lipid ontology, pathway, and network analysis with open access (Ni and Fedorova, 2020).

Unichem is a large scale data base of pointer between chemical structures and EMBL-EBI chemistry resources (https://www.ebi.ac.uk/unichem/), a module of connectivity search supports the conversion of identifiers.

Goslin is a very useful tool which allows the recognition/parsing and normalization of lipid names using different shorthand nomenclatures into a hierarchical structural representation that is then used to generate normalized lipid names following the lipid shorthand nomenclature (Kopczynski et al., 2022). The Goslin web application provides links to entries in SwissLipids and LIPID MAPS via these normalized names.

RefMet is a database of reference names for metabolites and lipids covering over 400,000 names. An API allows the conversion of names by querying the database online. For the conversion of identifiers tools like BridgeDb, Chemical Translation Service (CTS) or MetaboAnalyst can be used.

## How to map lipids onto metabolic networks?

The final goal is of course to link lipids to their biological function. Therefore a common strategy to interpret metabolomics or lipidomic data consists of locating them within the context of the whole metabolism and identifying the metabolic pathways they are mainly involved in, by using metabolic pathway databases such as KEGGS or genome-scale metabolic networks (GSMNs) and pathway overrepresentation approaches. However, the mapping onto metabolic pathways or GSMNs is much more tricky for lipids. Indeed, as mentioned above, lipids can be identified on different levels with different degrees of structural detail depending on the structural resolution. In parallel to this, metabolic pathway databases or GSMNs typically vary a lot in their details: they can, on the one side, contain lipids and lipid related molecules with full structural detail down to isomer level based on known biochemistry (in case of synthesis of lipid building blocks such as fatty acids or sphingoid bases), and on the other side, reactions and pathways for complex lipids are often only present with low structural details combined (e.g. only class-level compounds, such as for phospholipids which are often represented as PC pools of all the molecular species of PC, PI pools …). Furthermore, GSMNs use charged versions of metabolites and lipids as reactions are typically mass and charge balanced at the cytosolic pH of 7.3 and often focus on including the correct chemical formula, which is essential for balancing metabolic reactions, and take less care about correctly representing chemical entities (Witting, 2020). This adds complexity for the analysis of lipidomics data in the context of pathways as also differences in charge states between reported lipids and pathways need to be taken into account. When the analytical technique does not provide sufficiently detailed information about the lipid entities, then one single measured lipid entity might often match to multiple structures in pathway maps. On the contrary, for some lipid classes, GSMNs contain only generic nodes, such as phosphatidylcholines or triacylglycerols, to which basically all members of this lipid class, measured in a lipidomic experiment, would map. Automatic exact matching of lipids between measured entities and the corresponding species or classes represented in pathway maps or GSMNs is therefore limited by the lack of structural details provided on both sides. To make matters worse, identifiers are also different in that case. To overcome the discrepancy and still be able to map lipids to metabolic pathways or GSMNs, Poupin et al*.* developed a matching tool which uses the ChEBI ontology for matching measured lipids to lipids in a GSMN. This tool uses the relationship between two ChEBI identifiers as provided by the ChEBI ontology to relate precise lipid species to more generic lipid classes and acid/base related lipids. The further the two entries are separated in the ontology, the higher the retrieved matching distance will be (Poupin et al., 2020). They demonstrated that when matching the lipids of a large lipid database (with more than 900 species) on the human metabolic network, using only lipid names, they were able to only retrieve 3 out of the 968 lipids. This matching could be significantly improved by using the ChEBI identifiers, which were available for 73% of the lipids in the database: indeed, 54% of the database lipids could be matched to lipids in the metabolic network, either with an exact match or thanks to the ChEBI ontology. One drawback is that the coverage in the ChEBI ontology might be well-developed for certain lipids, but is missing for other cases. One suitable alternative might be the hierarchical classification system used by SwissLipids following the lipid nomenclature and shorthand rules that allows a more systematic mapping. Other possibilities to analyse links between lipid changes exist.

However, mapping to metabolic networks is one among the many possibilities for analysis of lipidomics data, focusing on metabolic pathways which are defined for the whole metabolism, and might not provide enough detailed interpretation as regard to specific lipid functions. Indeed, different lipids have different functions, which are very closely related to their structure (Gaud et al., 2021).

## Recommendations for the community

**Fig. 1:**
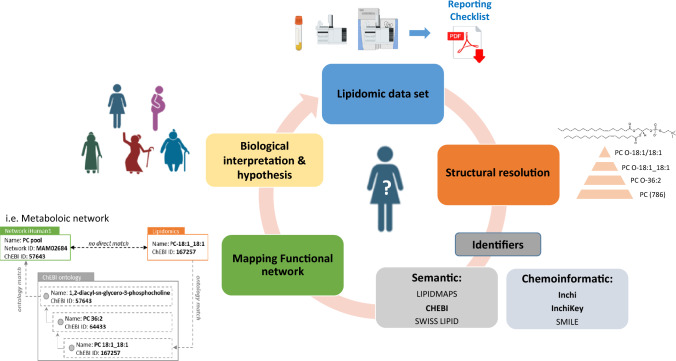
Essential elements that must be associated with a lipidomic data set

As we have just demonstrated, the correct naming and reporting of lipids identities remains complex and confusing, with many identifiers and initiatives available, which greatly complicates data sharing. While sharing of data for comparison and reuse become more evident, it also has become essential to harmonise practices in the field, especially for the reporting of lipid identities. To improve interoperability of lipidomic data sets we propose to take into account different points, as follows (Fig. 1):

- The dataset must be well characterised, including analytical conditions: this can be done through the Lipidomic reporting checklist: https://lipidomicstandards.org/reporting_checklist/ (McDonald et al., 2022).
- Structural resolution must be clear and use the naming conventions appropriately recommended by the international community (Liebisch et al., 2020).
- Recognised identifiers must be associated with the metabolites to ensure the measured lipids can be readable by a computer system.
- A ChEBI ID, SwissLipids ID. or LIPID MAPS ID should be associated with each measured lipid to allow the results to be interoperable and to facilitate mapping to metabolic networks (if available).
- Novel structures should be submitted to databases such as ChEBI and LIPID MAPS in order to be available to other scientists.
- All these identifiers have to be associated to data a set when it is stored in repositories such as Metabolights (https://www.ebi.ac.uk/metabolights/) or Metabolomics workbench (https://www.metabolomicsworkbench.org/).

In the future, lipids will be identified with more detail, which includes also sn-specificity and position and stereochemistry of double bonds. However, several further obstacles will still exist that need to be solved. First, lipids are synthesized and remodeled in different organelles, something that is currently not reflected in lipidomics results as typically entire tissues or cells are analyzed and sub-cellular location of lipids can therefore not be assessed. However, the use of structured databases and ontologies with controlled vocabularies allows linking of this information to lipid species stored in different databases. ChEBI uses special entries, which can be used to link a metabolite or lipid to organisms (e.g. CHEBI:78804 “Caenorhabditis elegans metabolite” or CHEBI:75771 “mouse metabolite”). This concept can be further expanded by adding tissue, cellular or sub-celluar specificity. As both SwissLipids and UniProtKB are based on ChEBI, future versions of SwissLipids will be able to leverage UniProtKB to build more extensive lipid libraries covering all taxa from UniProtKB, and sourcing annotations from UniProtKB to link all lipids to their metabolizing proteins.

In conclusion, it is important that lipids are identified and reported at the correct level, as technically supported by the employed analytical technique. This includes also the possibility to use identifiers from different databases and the different possibilities they offer. Several options for integration and interoperability, such as ID conversion services exist, enabling the cross-mapping between databases. The field must discipline itself to use the available resources and further develop them as well as educate the next generation of lipid scientists on the proper use of these resources.
